# Trade-off between Plasticity and Velocity in Mycelial Growth

**DOI:** 10.1128/mBio.03196-20

**Published:** 2021-03-16

**Authors:** Sayumi Fukuda, Riho Yamamoto, Naoki Yanagisawa, Naoki Takaya, Yoshikatsu Sato, Meritxell Riquelme, Norio Takeshita

**Affiliations:** a Microbiology Research Center for Sustainability (MiCS), Faculty of Life and Environmental Sciences, University of Tsukuba, Tsukuba, Ibaraki, Japan; b Institute of Transformative Bio-Molecules (WPI-ITbM), Nagoya University, Nagoya, Aichi, Japan; c Centro de Investigación Científica y de Educación Superior de Ensenada, CICESE, Ensenada, Baja California, Mexico; Universidad de Córdoba

**Keywords:** cell polarity, growth rate, microfluidic device, hyphae, filamentous fungi

## Abstract

Tip-growing fungal cells maintain cell polarity at the apical regions and elongate by *de novo* synthesis of the cell wall. Cell polarity and tip growth rate affect mycelial morphology. However, it remains unclear how both features act cooperatively to determine cell shape. Here, we investigated this relationship by analyzing hyphal tip growth of filamentous fungi growing inside extremely narrow 1 μm-width channels of microfluidic devices. Since the channels are much narrower than the diameter of hyphae, any hypha growing through the channel must adapt its morphology. Live-cell imaging analyses revealed that hyphae of some species continued growing through the channels, whereas hyphae of other species often ceased growing when passing through the channels, or had lost apical polarity after emerging from the other end of the channel. Fluorescence live-cell imaging analyses of the Spitzenkörper, a collection of secretory vesicles and polarity-related proteins at the hyphal tip, in Neurospora crassa indicates that hyphal tip growth requires a very delicate balance of ordered exocytosis to maintain polarity in spatially confined environments. We analyzed the mycelial growth of seven fungal species from different lineages, including phytopathogenic fungi. This comparative approach revealed that the growth defects induced by the channels were not correlated with their taxonomic classification or with the width of hyphae, but, rather, correlated with the hyphal elongation rate. This report indicates a trade-off between morphological plasticity and velocity in mycelial growth and serves to help understand fungal invasive growth into substrates or plant/animal cells, with direct impact on fungal biotechnology, ecology, and pathogenicity.

## Abstract (precis)

Tip-growing fungal cells maintain cell polarity at the apical regions and elongate by *de novo* synthesis of the cell wall. Cell polarity and tip growth rate affect mycelial morphology.

## INTRODUCTION

Cell morphology, which is controlled by polarity and growth, is fundamental for all cellular functions (1, 2). The core cell polarity machinery appears to be relatively conserved in animals, plants, and fungi (3, 4). First, polarity-signaling complexes assemble near a cell surface landmark, and locally assemble the cytoskeleton through actin or tubulin polymerization. Then, directed trafficking of vesicles and carriers contribute to local membrane and cell wall expansion. In addition, cell growth is controlled by turgor pressure, which drives the expansion of the cell cortex, especially in cell types covered by a cell wall (5, 6). Although both polarity and growth are essential for cell morphology, how growth speed and cell polarity cooperatively control cell shape remains unclear.

Filamentous fungi grow as highly polarized tubular cells that elongate through the continuous supply of membrane lipids and *de novo* synthesis of cell wall at the extending tip (7–11). The necessary proteins and lipids are delivered to the tip by vesicle trafficking via the actin and microtubule cytoskeletons and their corresponding motor proteins (12–16). The delivered secretory vesicles accumulate temporarily in an apical vesicle cluster, called the Spitzenkörper (SPK) (17–19). Vesicle exocytosis at the apical membrane allows release of secretory enzymes and the expansion of apical membrane and cell wall. Recent live-imaging analyses, including superresolution microscopy, have revealed that the multiple steps in polarized growth, such as the assembly of polarity markers, actin polymerization, and exocytosis, are temporally coordinated through pulsed Ca^2+^ influxes (20–22).

While the tip growth rate depends on the supply of vesicles, it has been reported that turgor pressure is also one of the driving forces of hyphal tip expansion (6). Turgor pressure in growing hyphae has been directly measured by using microinjection with pressure probes (23). Cytoplasmic bulk flow, which is evident in fast-growing fungi like Neurospora crassa, is also involved in the force to expand the hyphal tip (6, 24).

Microfluidic devices-based technology has been used to study the behavior of tip-growing plant cells (25–27) and, more recently, of filamentous fungi (28, 29). An elastic polydimethylsiloxane (PDMS) microfluidic device enabled measurement of the invasive pressure of tip-growing plant pollen tubes (30). Likewise, scanning probe microscopy (SPM), with a sensor probe that directly indents the cellular surface, is available for measurement of cellular stiffness in a noninvasive manner (31). These methods, in combination with cell biological approaches, are powerful tools to investigate mechanical properties in living cells.

**FIG 1:**
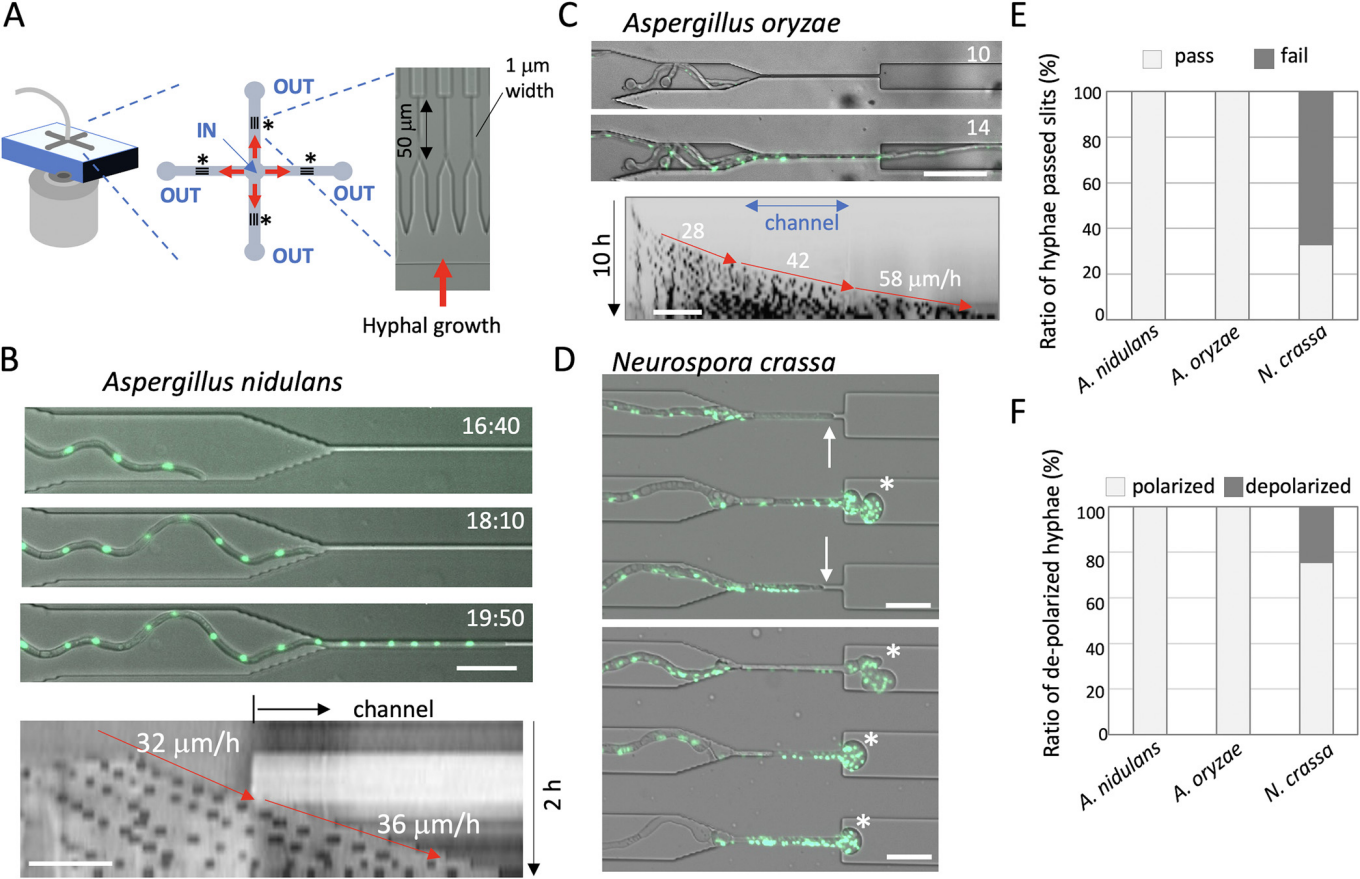
A. nidulans and A. oryzae but not N. crassa hyphae pass through the channels. (A) Design of the microfluidic device, with inflow at the center (IN) and outflows at the four path ends (OUT). Twenty microchannels of 1 μm width and 50 μm length were included between the inlet and outlet for each arm (asterisks) of the cross-shaped design. (B, upper) Time series showing a hypha of A. nidulans (nuclei labeled with GFP) growing into the channel. The elapsed time is given in hours:minutes. (B, lower) Kymograph along the growth axis before and in the channel (from Movie S1 in the supplemental material). The hyphal elongation rates before and in the channel are shown by arrows. Total 2 h; scale bar 20 μm. (C, upper) Time series showing a hypha of A. oryzae (nuclei labeled with GFP) passing through the channel. The elapsed time is given in hours. (C, lower) Kymograph along the growth axis before, in, and after the channel (from Movie S2). Total 10 h; scale bar 20 μm. (D) Images of N. crassa (nuclei labeled with GFP) hyphae that have stopped growing in the channels (arrows) and depolarized hyphae exiting from the channels (asterisks) (from Movie S3). Scale bar 20 μm. (E) Ratio of the hyphae that successfully passed through the channels (pass) or stopped in or exiting from the channels (fail) in A. nidulans, A. oryzae, and N. crassa; *n* = 50 for each. (F) Ratio of polarized or depolarized hyphae that passed through the channels in A. nidulans, A. oryzae, and N. crassa; *n* = 50 for each.

Here, we constructed a microfluidic device with 1 μm-width channels, which are narrower than the diameter of fungal hyphae, and observed growth as hyphae grew into, through, and out of the channels (Fig. 1A). The present study aimed to identify the relationship between cell polarity and growth rate by observing the forced morphological changes of growing hyphae under a microscope. Our results will help to understand fungal invasive growth into substrates or host plant/animal cells, which knowledge can be applied to the fields of fungal biotechnology, ecology, and pathogenicity.

## RESULTS

### Aspergillus nidulans and A. oryzae, but not N. crassa, hyphae grow through the channels.

The PDMS microfluidic device used in this study possesses multiple micro channels, 1 μm wide and 50 or 100 μm long (Fig. 1A). Fungal spores were inoculated to the inlet at the center of the device (IN). The medium solution was continuously supplied to the inlet with the help of a pump (0.8 μl per hour) and flowed out from the four outlet corners (OUT).

We monitored hyphal growth of Aspergillus nidulans as it grew into, through, and out of the channels. We used a strain whose nuclei were visualized by fluorescence of the nuclear localization signal of the transcription factor StuA tagged with green fluorescent protein (GFP) (32). The hyphal widths were 2 to 3 μm before entering the channel under this condition. All observed hyphae grew into the channels, passed through them, and continued to grow (50 > *n*) (Fig. 1B, Fig. S1A, Movie S1 in the supplemental material). The kymograph along the growth axis indicated comparable growth rates of 37 ± 15 μm/h (*n* = 20) before, through (Fig. 1B), and after the channels (Fig. S1A). In some cases, two or three hyphae passed through the same channel (Fig. S1B, Movie S1). In the same way, we tested Aspergillus oryzae, which is an important species for traditional food fermentation and modern biotechnology (33). We used a strain in which histone H2B is fused with GFP (34). Again, all observed hyphae went into the channels, passed through, and continued to grow after emerging from the other end without decreased growth rates (84 ± 37 μm/h, *n* = 30) (Fig. 1C, Movie S2).

10.1128/mBio.03196-20.1 FIG S1 (A to F) A. nidulans but not N. crassa hyphae pass through the channels. (A) Time series showing a hypha of A. nidulans (nuclei labeled with GFP) growing into the channel. Kymograph along the growth axis before, in, and after the channel (from Movie S1). The hyphal elongation rates are shown by an arrow. Total 6 h, scale bar: 20 μm. (B) Time series of A. nidulans (nuclei labeled with GFP) two or three hyphae passing through the same channel (see Movie S1). Each hyphal tip is shown by arrows. Scale bars: 20 μm. (C) Image of the N. crassa spores germinated to the opposite side of slits. (D) Ratio of direction in germination toward or opposite to channels in A. nidulans, A. oryzae, and N. crassa; *n* = 50 each. (E) Time series images of N. crassa (DIC, left; CHS-1-GFP, right) hyphae growing into a channel (from Movie S8). The arrows indicate the SPK. The elapsed time is given in minutes. Scale bar: 20 μm. (F) The plot profile along the apical membrane indicates the signal peaks of SPK (arrows) at 10, 20, and 30 min, but not at 40 or 50 min. The plot profile along the growth axis (right) indicates the peaks at the apex of hyphae at 10, 20, or 30 min, but at the subapex at 40 or 50 min. (G to J) Increased number of septa and SPK. (G and H) Image sequence of forming septa (arrows) and formed septa (arrow heads) in the depolarized hyphae through the channel (G) and in the hyphae through the channel (H). The elapsed time is given in hours:minutes. Scale bar: 20 μm. (I) Number of septa in 200-μm hyphae around the channel, in the hyphae that passed the channels, or in the depolarized hyphae. Error bars = SD; *n* = 5; ***, *P* ≤ 0.001. (J) SPK of N. crassa hypha grown in MM + 0.6 M KCl. Images of the N. crassa (SPK labeled with GFP) hypha in the channel from Movie S8. The arrow indicates the SPK before, in, and after the channel. The elapsed time is given in hours:minutes. Scale bar: 20 μm. (K to O) Phylogenetic tree and growth on the plates. (K) Phylogenic tree of filamentous fungi used in this study. Maximum likelihood (ML) tree obtained from the ITS1 and ITS2 regions of the fungal strains. The bootstrap consensus inferred from 100 replicates. (L) Correlation between the hyphal elongation rate and depolarized hyphae. Two groups are shown by red or blue ellipses. (M) No correlation between the hyphal width with the growth defect in channels. (N) Colony diameter of N. crassa, *R. oryzae*, and *C. cinerea* on MM or MM + 0.6 M KCl plates. (O) Colonies of A. nidulans (An), A. oryzae (Ao), *C. cinerea* (Cc), N. crassa (Nc), and *R. oryzae* (Ro) growth on minimal medium (MM) plates or MM + 0.6 M KCl plates for 2 to 7 days. (P and Q) Elastic modules measurement by a scanning probe microscope (SPM). The principle of SPM equipment is composed of the following: (i) laser diode and photo detector; (ii) cantilever and holder; and (iii) scanner (http://www.shimadzu.com/an/surface/spm/faq/index.html). The basis of the force curve measurement is the measurement performed at one point of the sample. As the distance of the probe changes relative to the sample, this distance can be plotted on the horizontal axis, as shown on the graph. Also, it is possible to calculate from the spring constant of the cantilever and plot this on the vertical axis as nN. When the probe and sample distance are far away, the force does not work, hence the vertical axis is ①. When the cantilever touches the sample it is ②. After that, the slope of the graph when the repulsive force acts reflects the hardness of the sample, shown as ③. When a release-curve is observed, often a large attractive area can be seen. This is because the probe is caught by the adsorption layer on the sample surface, shown as ④. From this approach-curve and release-curve, Young’s modulus can be calculated using JKR or Hertz. Therefore, by saving the data at each pixel, a mapping image can be constructed. Fungal cells are grown in a noninvasive manner at high magnifications. Download FIG S1, TIF file, 2.7 MB. Copyright © 2021 Fukuda et al. 2021 Fukuda et al. https://creativecommons.org/licenses/by/4.0/ This content is distributed under the terms of the Creative Commons Attribution 4.0 International license.

10.1128/mBio.03196-20.3 MOVIE S1 *Aspergillus nidulans* strain whose nuclei were visualized by GFP grew into, through, and out of the channels. Every 10 min, total 10 h, scale bar: 20 μm. Download Movie S1, AVI file, 2.6 MB. Copyright © 2021 Fukuda et al. 2021 Fukuda et al. https://creativecommons.org/licenses/by/4.0/ This content is distributed under the terms of the Creative Commons Attribution 4.0 International license.

10.1128/mBio.03196-20.4 MOVIE S2 *Aspergillus oryzae* strain whose nuclei were visualized by GFP grew into, through, and out of the channels. Every 20 min, total 14 h, scale bar: 50 μm. Download Movie S2, AVI file, 0.9 MB. Copyright © 2021 Fukuda et al. 2021 Fukuda et al. https://creativecommons.org/licenses/by/4.0/ This content is distributed under the terms of the Creative Commons Attribution 4.0 International license.

We examined another model filamentous fungus, Neurospora crassa, whose hyphae usually grow faster and have a larger diameter than those of A. nidulans (7, 35) (see below). We used a strain in which histone H1 is fused with GFP (24). Some hyphae penetrated into the channels, but often their growth speed slowed down and stopped before reaching the end of the channel (Fig. 1D arrows, Movie S3). The hyphae that passed through the channels frequently lost tip polarization and started to swell (Fig. 1D asterisks, Movie S3). The depolarized hyphae stopped growing after a while (2 to 10 h), then lost the nuclear GFP signal (Movie S3). The growth arrest inside the channels and the loss of polarity of the hyphae after exiting the channels were characteristic of N. crassa and were never observed for A. nidulans or A. oryzae (Fig. 1E and F). In N. crassa, 33% of the hyphae grew out of the channel without losing apical polarity (Fig. 1E) (*n* = 50). In addition, N. crassa spores that were trapped in front of the channels frequently germinated toward the opposite side of channels, i.e., did not enter the channel, (87%, Fig. S1C). This phenomenon was observed less frequently in A. nidulans or A. oryzae, with percentages of 53 and 64%, respectively (Fig. S1D).

10.1128/mBio.03196-20.5 MOVIE S3 *Neurospora crassa* strain whose nuclei were visualized by GFP often showed depolarized hyphae out of the channels. Every 5 min, total 20 h, scale bar: 20 or 50 μm. Download Movie S3, AVI file, 18.1 MB. Copyright © 2021 Fukuda et al. 2021 Fukuda et al. https://creativecommons.org/licenses/by/4.0/ This content is distributed under the terms of the Creative Commons Attribution 4.0 International license.

### Cell polarity loss after forced morphological changes in N. crassa.

**FIG 2:**
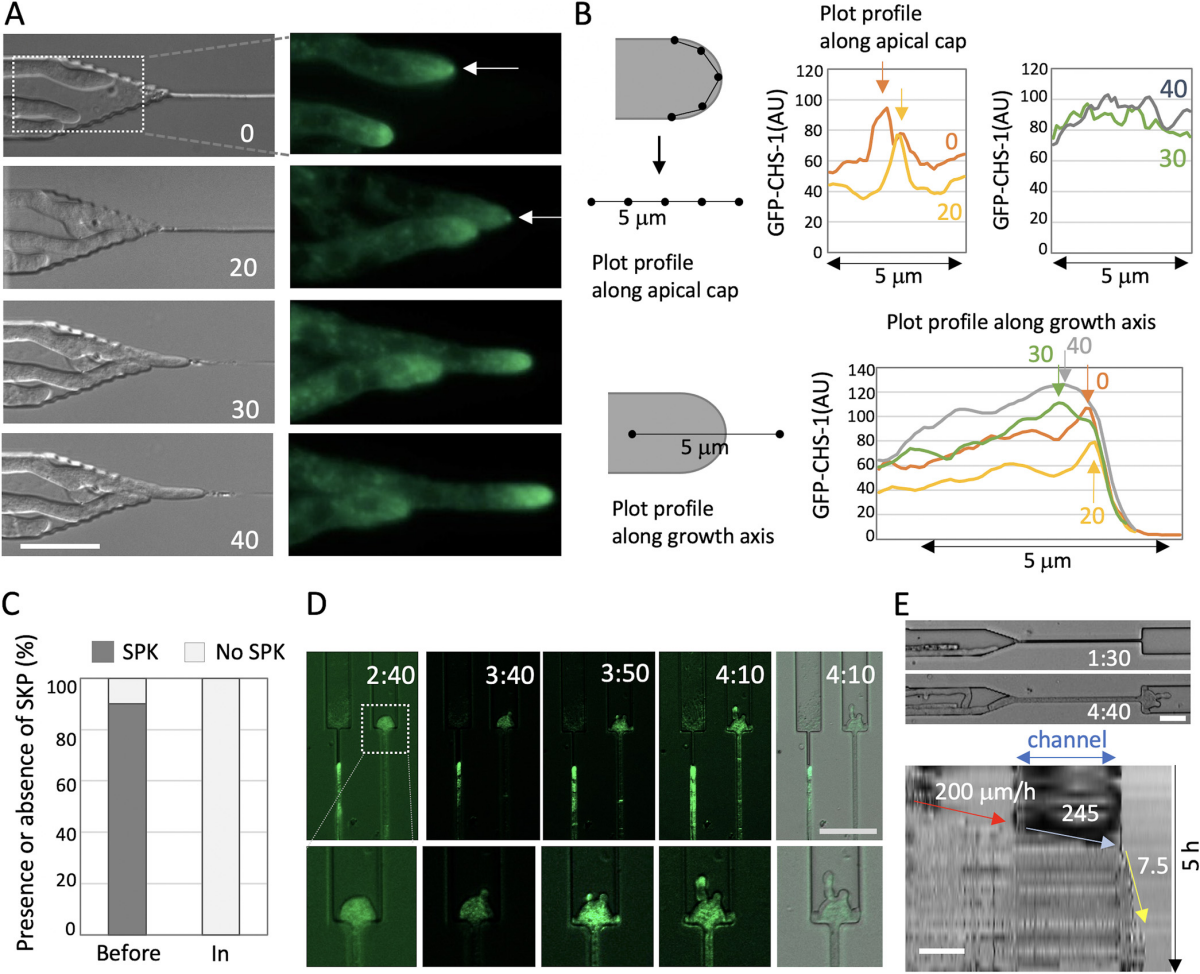
SPK localization during confined growth in N. crassa. (A) Time series images of N. crassa (DIC, left; CHS-1-GFP, right) hyphae growing into a channel (from Movie S4). The arrows indicate the SPK. The elapsed time is given in minutes. Scale bar 20 μm. (B) Scheme to measure GFP signal intensity along the apical membrane (upper) or the growth axis (lower). The plot profile along the apical membrane (upper) indicates the signal intensity peaks of the SPK (arrows) at 0 or 20 min, but not at 30 or 40 min. The plot profile along the growth axis (lower) indicates the peaks at the apex of hyphae at 0 or 20 min, but at the subapex at 30 or 40 min. (C) Ratio of presence or absence of SPK in hyphae before or in channels; *n* = 20 or 10, respectively. (D) Image sequence of the depolarized hypha after exiting the channel in the N. crassa (CHS-1- GFP) hyphae (from Movie S5). The elapsed time is given in hours:minutes; scale bar 50 μm. (E) Kymographs along the growth axis of the channel from Movie S5. The hyphal elongation rates before entering, through the channel, and after exiting the channel are shown by arrows. Total 5 h; scale bar 50 μm.

We investigated the cell polarity in N. crassa hyphae growing in the channels by monitoring GFP-tagged chitin synthase class III (CHS-1) at the SPK (36). Accumulation of GFP-CHS-1 at the SPK was clearly observed at the tips of growing hyphae before growing into the channels (Fig. 2A, Fig. S1E, Movie S4). The hyphae entered the channels and then stopped growing, coinciding with a loss of the GFP signal at the SPK and dispersion of the fluorescence signal along the cytoplasm of the tip region, with high intensity level of GFP (Fig. 2A and B, Fig. S1E and F). Distinct accumulation of GFP-CHS-1 at the SPK was hardly observed in those hyphae inside the channels (Fig. 2C). In the depolarized swollen hyphae exiting a channel, the fluorescence signal was diffused and weak, but became visible again at the apex of the multiple branches that formed when polarized growth resumed after channel exit (Fig. 2D arrows, Movie S5). The kymograph along the growth axis indicated comparable growth rate before and in the channels, 200 and 245 μm/h; however, the hyphae drastically decreased the growth rate after exiting the channel (Fig. 2E).

10.1128/mBio.03196-20.6 MOVIE S4 *Neurospora crassa* strain expressing GFP-CHS-1 penetrated into the channels and then stopped growing. Every 10 min, total 50 min, scale bar: 20 μm. Download Movie S4, AVI file, 0.5 MB. Copyright © 2021 Fukuda et al. 2021 Fukuda et al. https://creativecommons.org/licenses/by/4.0/ This content is distributed under the terms of the Creative Commons Attribution 4.0 International license.

10.1128/mBio.03196-20.7 MOVIE S5 *Neurospora crassa* strain expressing GFP-CHS-1 penetrated into the channels and then showed depolarized hyphae out of the channels. Every 10 min, total 6 h, scale bar: 20 μm. Download Movie S5, AVI file, 1 MB. Copyright © 2021 Fukuda et al. 2021 Fukuda et al. https://creativecommons.org/licenses/by/4.0/ This content is distributed under the terms of the Creative Commons Attribution 4.0 International license.

CHS-1-GFP is also known to localize at septa during their formation (36). We found that the depolarized hyphae possessed two-times more septa within the narrow channels than the hyphae that successfully passed through the channels without presenting polarity defects (Fig. S1G to I), suggesting that the cell cycle progresses and deposition of cross walls continues when tip growth is inhibited.

### Relationship between growth rate and polarity maintenance.

**FIG 3:**
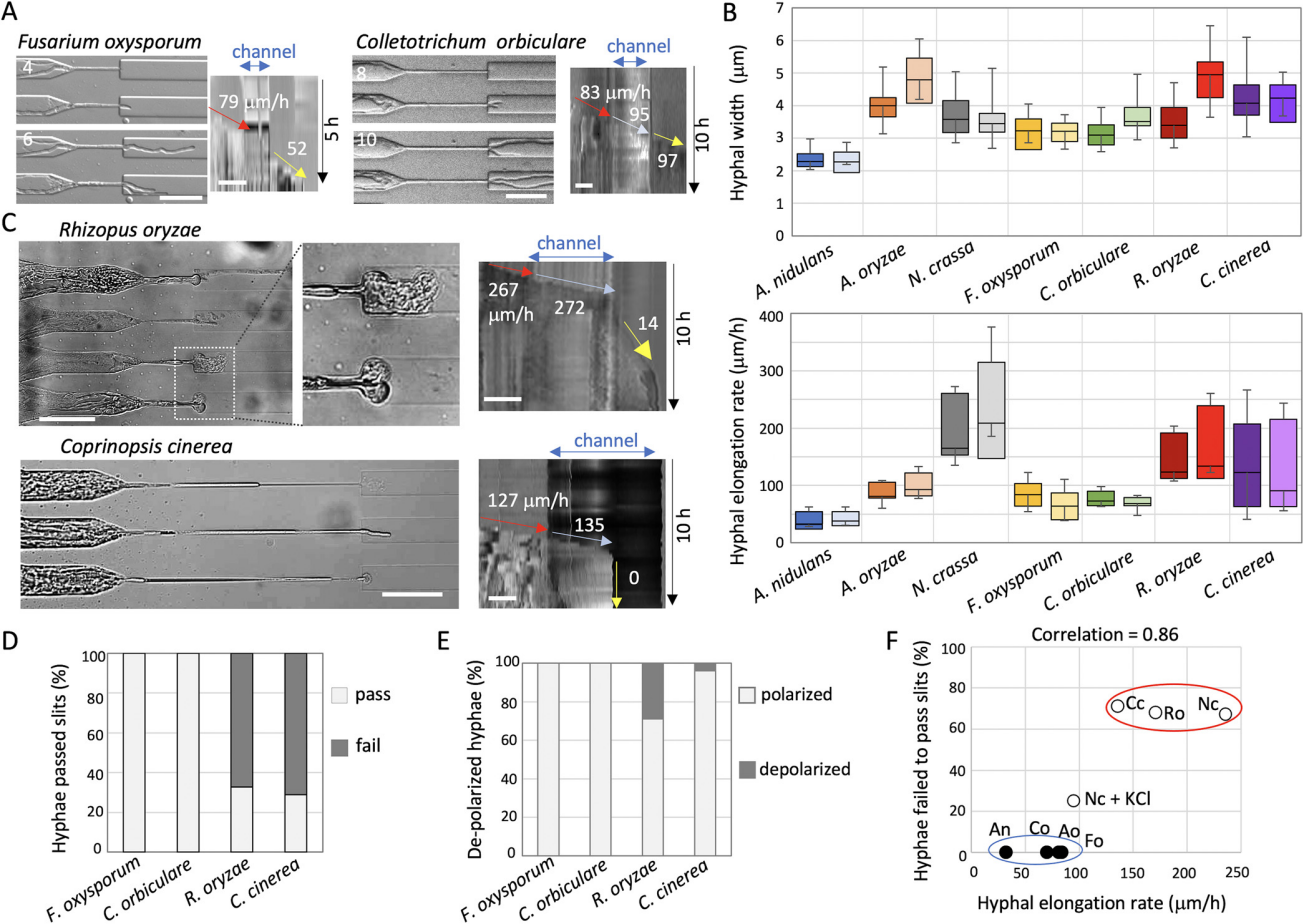
Relationships among hyphal width, growth rate, and polarity maintenance. (A) Time series of F. oxysporum (left) and *C. orbiculare* (right) hyphae that passed through the channel (from Movie S6). The elapsed time is given in hours. Kymographs along the growth axis of the channel from Movie S6. The hyphal elongation rates before entering and after exiting the channel are shown by arrows. Total 5 h (left) and 10 h (right); scale bar 50 μm. (B) Boxplots of hyphal width (upper) and hyphal elongation rate (lower) in A. nidulans, A. oryzae, N. crassa, F. oxysporum, *C. orbiculare*, *R. oryzae*, and *C. cinerea* before entering the channels (darker colors) and after exiting the channels (lighter colors); *n* = 26, 40, 80, 53, 45, 14, and 20, respectively (upper), *n* = 20 (lower). Depolarized hyphae were not counted. (C) Images of depolarized hyphae of *R. oryzae* (upper) and of *C. cinerea* hyphae that stopped growing in the channel or after exiting the channel (lower), from Movie S7. Kymograph along the growth axis of the channel. Total 10 h; scale bar 50 μm. (D and E) Ratio of the hyphae that successfully passed through the channel (pass) or stopped in or just after exiting the channels (fail) (D), and the ratio of depolarized hyphae after exiting the channels (E) in F. oxysporum, *C. orbiculare*, *R. oryzae*, and *C. cinerea*; *n* = 29, 20, 52, and 52, respectively. (F) Correlation between the hyphal elongation rate with the growth defect in channels. Two groups are shown by red or blue ellipses.

We tested two plant-pathogenic fungi, Fusarium oxysporum and Colletotrichum orbiculare (37, 38), using the same microfluidic devices. Since plant-pathogenic fungal hyphae have to penetrate the space between tightly connected plant cells, polarity maintenance in spatially confined growth should be important for their pathogenicity. Almost all hyphae of F. oxysporum and *C. orbiculare* grew into and passed through the channels while maintaining their growth rates (83 ± 27 μm/h and 91 ± 16 μm/h, *n* = 53 and 45, respectively) (Fig. 3A, Movie S6).

10.1128/mBio.03196-20.8 MOVIE S6 *Fusarium oxysporum* strain grew into, through, and out of the channels. Every 20 min, total 7 h, scale bar: 50 μm. *Colletotrichum orbiculare* strain grew into, through, and out of the channels. Every 20 min, total 16 h, scale bar: 50 μm. Download Movie S6, AVI file, 4.2 MB. Copyright © 2021 Fukuda et al. 2021 Fukuda et al. https://creativecommons.org/licenses/by/4.0/ This content is distributed under the terms of the Creative Commons Attribution 4.0 International license.

To investigate the reason why only N. crassa but not the other fungi showed growth defects during spatially confined growth, we compared the widths of hyphae and hyphal elongation rates of all fungi growing in the device (Fig. 3B). The results corresponding to before entering and after exiting the channels are shown in dark and bright colors, respectively. The hyphal widths of A. nidulans were 2 to 3 μm, whereas those of N. crassa, F. oxysporum, and *C. orbiculare* were 3 to 4 μm, and those of A. oryzae were slightly wider. These results suggest that the hyphal widths are not correlated with the growth defect shown in the channels. There was no significant difference in the hyphal widths between before entering and after exiting the channels except with A. oryzae. It is known that A. oryzae increases hyphal width as cultivation time passes (34). Since the widths in mature hyphae of N. crassa are known to be over 10 μm, the hyphae we observed under this condition were considered young hyphae.

In contrast, the average hyphal elongation rate measured in A. nidulans was less than 50 μm/h, whereas those in A. oryzae, F. oxysporum, and *C. orbiculare* were 50 to 100 μm/h (Fig. 3B, lower graph). Notably, the average hyphal elongation rate measured in N. crassa was 150 to 250 μm/h, considerably higher than that of the other fungi.

To examine the relationship between growth rate and growth defect inside the channels, we further examined Rhizopus oryzae and Coprinopsis cinerea dikaryon, whose hyphal elongation rates are known to be relatively high (39, 40). The hyphal elongation rates of *R. oryzae* and *C. cinerea* in the device were 100 to 250 μm/h (Fig. 3B), whereas the hyphal widths were 3 to 5 μm, indicating that these two fungi grow faster than the other fungi, and similar to N. crassa. At least one hypha entered one channel, however, hyphae of *R. oryzae* and *C. cinerea* often stopped growing in or shortly after exiting the channels (Fig. 3C and D, Movie S7). Depolarized hyphae were sometimes observed after exiting the channels in *R. oryzae*, similarly to what was observed for N. crassa (Fig. 3C and E). We tested various fungal species of different phylogenetic lineages (41). However, the observed output did not correlate with the phylogenetic distance (Fig. S1K). Altogether, these results indicated that neither phylogenetic relevance, nor the width of hyphae, are correlated with the growth defects seen inside the channels. In contrast, the hyphal elongation rate displayed a strong correlation with observed growth defects inside the channels (Fig. 3F, correlation: 0.86, Fig. S1L and M).

10.1128/mBio.03196-20.9 MOVIE S7 *Rhizopus oryzae* strain showed depolarized hyphae out of the channels. Every 10 min, total 15 h, scale bar: 50 μm. *Coprinopsis cinerea* dikaryon strain penetrated into the channels and then stopped growing. Every 10 min, total 24 h, scale bar: 50 μm. Download Movie S7, AVI file, 9.3 MB. Copyright © 2021 Fukuda et al. 2021 Fukuda et al. https://creativecommons.org/licenses/by/4.0/ This content is distributed under the terms of the Creative Commons Attribution 4.0 International license.

### Contribution of turgor pressure for polarity maintenance.

**FIG 4:**
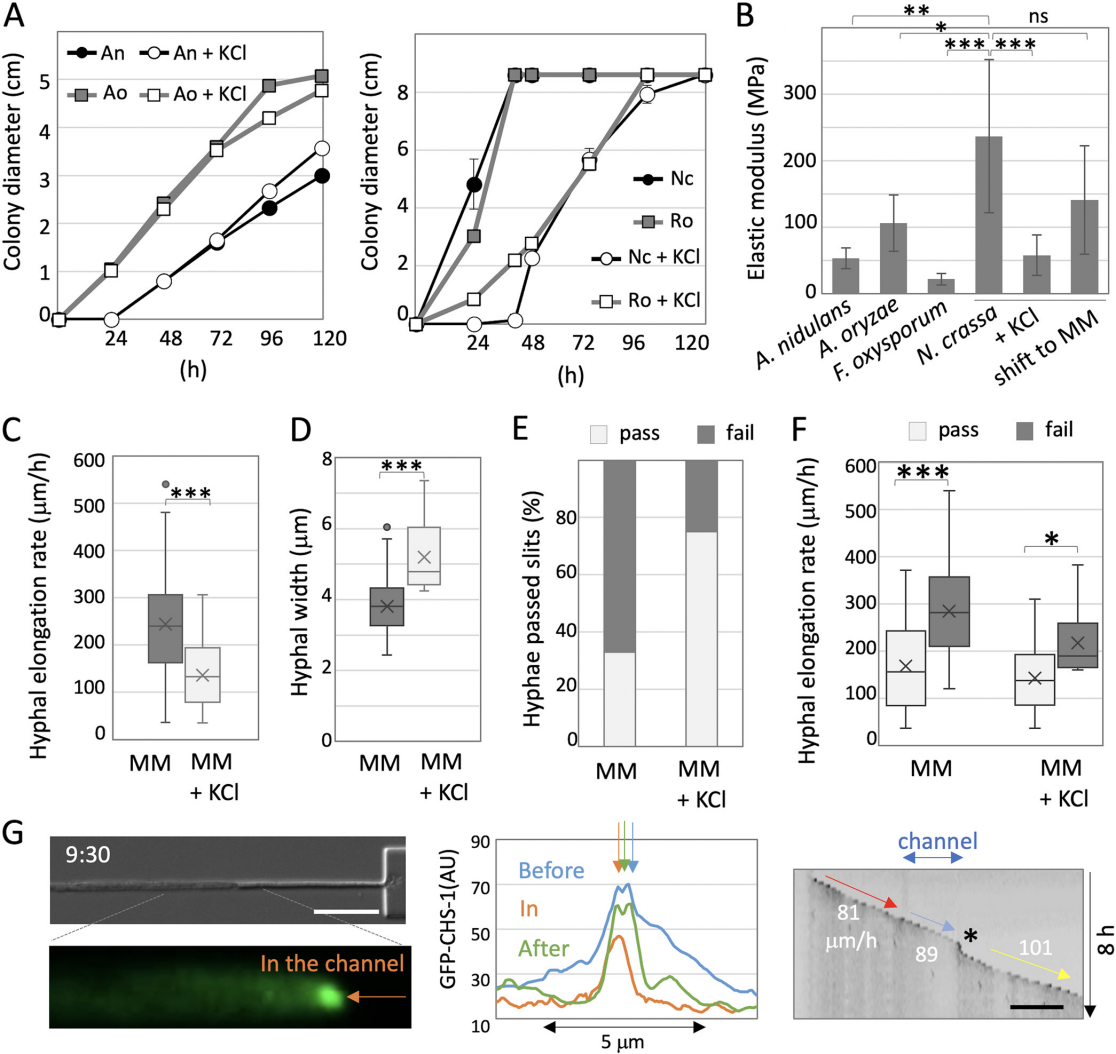
Contribution of growth rate for polarity maintenance. (A) Colony diameter of A. nidulans and A. oryzae (left), N. crassa and *R. oryzae* (right) on minimal medium (MM) or MM + 0.6 M KCl plates. (B) Elastic modulus measured by a scanning probe microscope in the hyphae of A. nidulans, A. oryzae, F. oxysporum, and N. crassa grown in MM. In addition, N. crassa grown in MM + 0.6 M KCl, and shifted to MM. Error bars represent standard deviations (SD); *n* = 3 to 10 in 1 to 3 hyphae; ***, *P* ≤ 0.001; **, *P* ≤ 0.01; *, *P* ≤ 0.05; ns, not significant. (C) Hyphal elongation rate of N. crassa hyphae grown in MM and MM + 0.6 M KCl. Error bars represent SD; *n* = 20; ***, *P* ≤ 0.001. (D) Hyphal width of N. crassa grown in MM and MM + 0.6 M KCl. Error bars represent SD; *n* = 20; ***, *P* ≤ 0.001. (E) Ratio of the hyphae that successfully passed through the channel (pass) or stopped within or just after exiting the channels (fail) in N. crassa grown in MM or MM + 0.6 M KCl; *n* = 53 and 32, respectively. (F) Boxplots of hyphal elongation rate in N. crassa hyphae just before entering the channel, pass or fail, grown in MM or MM + 0.6 M KCl; *n* = 18, 33, 18, and 9, respectively; ***, *P* ≤ 0.001; *, *P* ≤ 0.05. (G, left) Image of an N. crassa hypha (SPK labeled with GFP) growing within the channel in MM + 0.6 M KCl (from Movie S8). The arrow indicates the SPK. The elapsed time is given in hours:minutes; scale bar 20 μm. (G, middle) Plot profile along the apical membrane indicates the signal peaks of SPK (arrows) before, in, and after the channel(see Fig. S1J). (G, right) Kymograph of GFP signal along the growth axis (from Movie S8). Total 8 h; scale bar 100 μm. The hyphal elongation rates before entering and after exiting the channel are shown by arrows.

Why do hyphae of N. crassa, *R. oryzae*, and *C. cinerea* generally grow faster than those of A. nidulans and other species? One possibility points to the fact that N. crassa, *R. oryzae*, and *C. cinerea* hyphae have higher turgor pressure. This is supported by the results showing they were sensitive to the high osmotic condition generated by addition of 0.6 M KCl, resulting in decrease of turgor pressure (Fig. 4A and Fig. S1N and O). In contrast, A. nidulans, A. oryzae, and F. oxysporum were not sensitive to the high osmotic condition.

Indeed, we measured the elastic modulus, which represents forces balanced in the opposite direction of turgor pressure, by using a scanning probe microscope (SPM). The SPM scans sample surfaces with an extremely sharp sensor probe and measures the physical property of fungal cells in a noninvasive manner at high magnifications (Fig. S1P and Q). The elastic moduli of N. crassa hyphae, 236 ± 115 MPa, were significantly higher than those of A. nidulans, A. oryzae, and F. oxysporum (Fig. 4B), reflecting that the turgor pressure in N. crassa is higher. The elastic moduli of N. crassa decreased to 58 ± 30 MPa in the hyphae grown in minimal medium (MM) with 0.6 M KCl (Fig. 4B), suggesting that the turgor pressure decreased under high osmotic condition. After the hyphae grown in MM + 0.6 M KCl were shifted to MM for 30 min, the elastic moduli recovered to 141 ± 82 MPa.

In order to decrease the turgor pressure in hyphae of N. crassa grown inside the device, N. crassa was grown under high osmotic condition attained with 0.6 M KCl. The hyphal elongation rate just before the channels decreased in high osmotic condition from 239 ± 50 to 151 ± 21 μm/h (Fig. 4C), whereas the hyphal widths increased from 3.8 ± 0.7 to 5.2 ± 1.0 μm (Fig. 4D). Notably, the ratio of hyphae that passed through the channels increased from 33% to 75% in the high osmotic condition (Fig. 4E), which is correlated with the decreased hyphal elongation rate (Fig. 3F, Nc + KCl). Although 25% of the hyphae still stopped growing in the channels, depolarized hyphae were not observed (Fig. S1L). We compared the hyphal elongation rate just before the channels in the hyphae that passed or failed to pass, and found that hyphal elongation rate was lower in the hyphae that passed the channels than seen in hyphae which failed to pass in normal and high osmotic conditions (Fig. 4F).

**FIG 5:**
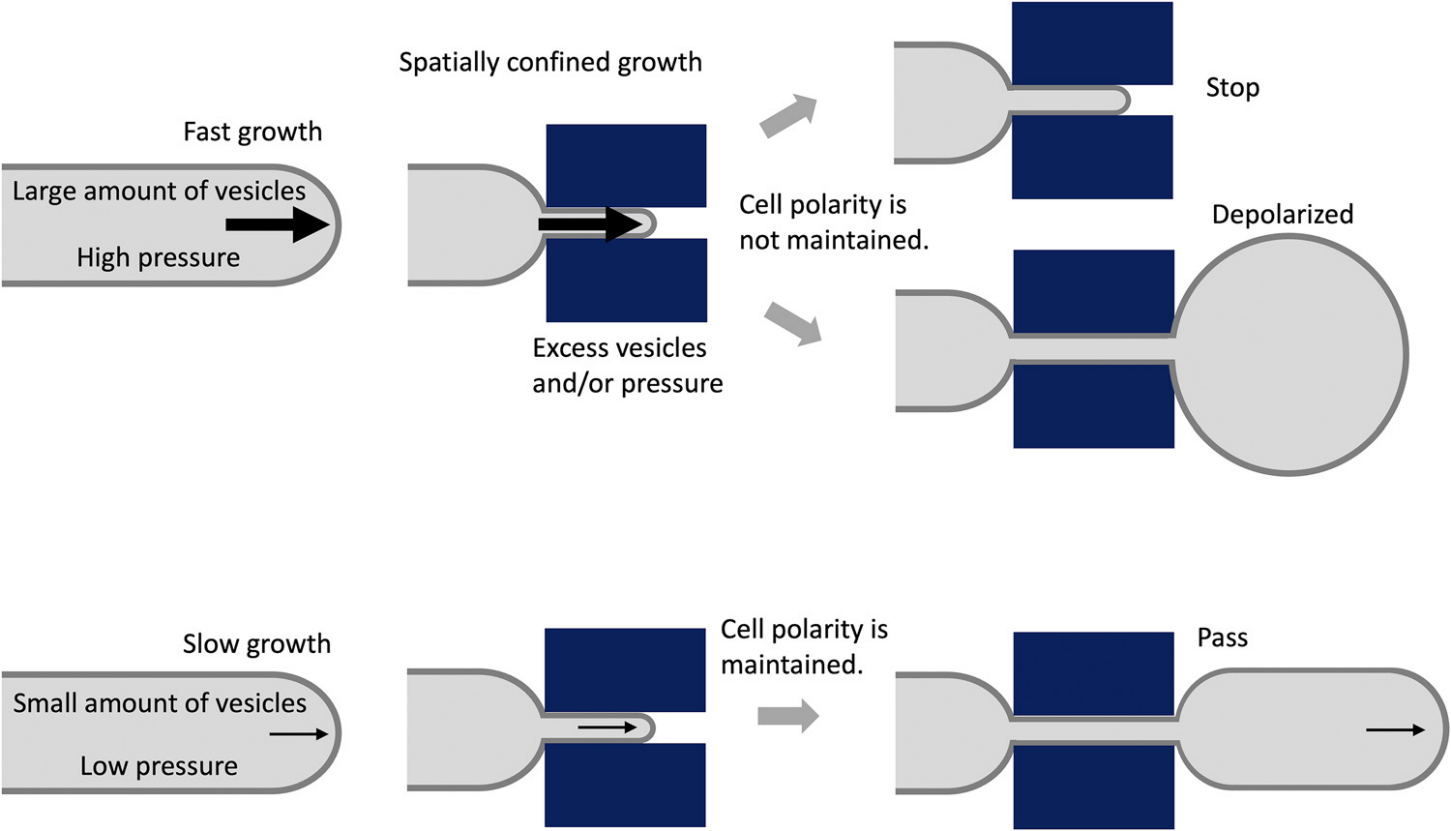
Relationship between extension rate and the ability to adapt to spatially confined growth. Cartoon representation of trade-off between cell plasticity and growth rate in spatially confined growth, and how they are correlated and act cooperatively to determine cell shape. When fast-growing hyphae pass through a narrow channel, a massive number of vesicles are forced to be congregated and mislocalized at sites others than the tip region, resulting in the depolarized growth and tip swelling when exiting the channels. In addition, a high turgor pressure makes all the machinery collect isotropically at the tip in an uncontrolled manner, thus generating a swollen tip. In contrast, a lower growth rate allows hyphae to maintain positioning of the cell polarity machinery, thereby permitting growth in highly confined spaces.

Under high osmotic conditions, the SPK labeled by GFP-CHS-1 was clearly observed at the tips of growing hyphae even in the channels (Fig. 4G, Fig. S1J, Movie S8). Although hyphae swelled slightly when exiting the channel (Fig. 4G right, asterisk, Movie S8), they grew into and passed through the channels while maintaining their growth rates (Fig. 4G right, arrows). These results indicate that the growth rate is important for the maintenance of cell polarity in spatially confined growth derived from passing through the channels (Fig. 5).

10.1128/mBio.03196-20.10 MOVIE S8 *Neurospora crassa* strain expressing GFP-CHS-1 grew into, through, and out of the channels in the high osmotic condition. Every 10 min, total 15 h, scale bar: 50 μm. Download Movie S8, AVI file, 2.8 MB. Copyright © 2021 Fukuda et al. 2021 Fukuda et al. https://creativecommons.org/licenses/by/4.0/ This content is distributed under the terms of the Creative Commons Attribution 4.0 International license.

## DISCUSSION

This study showed that hyphae from several fungal species of different phylogenetic lineages were able to grow into microchannels narrower than their width, as described before for plant tip-growing cells (27). Microfluidic devices have also been used recently to study the hyphal growth of filamentous fungi (28, 29, 42, 43). It was first found that hyphae of N. crassa, *R. oryzae*, and *C. cinerea* either ceased growing when passing through the channels or lost polarity upon exiting the channels. The observed effects did not correlate with their taxonomic classification or with the width of hyphae, but correlated with the hyphal elongation rate. Fast-growing fungi possess the advantage of quickly covering new nutrient-rich substrates or free open spaces. However, at the same time, they may lack the ability to regulate cell shape properly when growing in spatially confined environments. As far as we know, this is the first report indicating a trade-off between growth rate and morphological plasticity, which suggests the significance of slow growth for the cooperative control of cell polarity and cell growth. This characteristic is considered a case of convergent evolution, given that each fungus possesses a similar morphology and physiology adapted to different environmental factors despite that they are phylogenetically distant. It will be fascinating in the near future to study whether a similar relationship is observed in other tip-growing cells, such as pollen tubes and root hairs of different plant species.

Our results indicate that hyphal tip growth requires a very delicate balance of ordered exocytosis to maintain polarity under spatially constrained circumstances. In fast growing hyphae, such as N. crassa, a large number of secretory vesicles per time unit are presumably supplied to the hyphal tips, resulting in a conspicuous SPK (44). When fast growing hyphae enter into the narrow channels, a massive number of vesicles are forced to congregate in the tip region. The cytoplasmic space in those squeezed hyphae is probably too small for all the secretory vesicles to fit within the tip region. The space constraints therefore cause the excess of secretory vesicles to mislocalize at sites others than the tip region, resulting in loss of apical polarity and tip swelling when exiting the channels (Fig. 5). In fact, the lack of localization of some vesicular markers such as CHS-1 at the tip, as well as the dispersed fluorescence observed instead when N. crassa hyphae grew through the channels, supports the idea than an excess of vesicles accumulates in a nonorganized manner at the subapical region. When hyphae are forced to grow through a very narrow channel, under a high turgor pressure yet maintaining the same growth speed, large portions of the cell wall building machinery accumulate at the subapical region. Upon exiting the channel, all the machinery gets incorporated in an uncontrolled manner at the tip, resulting in isotropic growth and generating a swollen tip. This causes several new polarity axes to become established, such that growth resumes in the form of multiple branches. In the case of A. nidulans, even if hyphae are squeezed when entering the channel, the vesicles, presumably less abundant, manage to maintain their flow rate, spacing, and movement, and hence growth remains unaffected during passage and upon exiting the channel (Fig. 5).

Filamentous fungi play a major role in degradation of biopolymers found in nature for organic material recycling (45, 46). Some fungi are useful in biotechnology and traditional food fermentation (33, 47), where solid-state cultivation is especially important (48). Hyphal invasive growth into host cells (plant or animal) is essential for pathogenicity and for symbiosis (37, 49, 50). Our results help understand the mechanisms of fungal invasive growth into substrates or host cells by spatially confined growth and how cell morphology is controlled by cell polarity and cell growth, and are thus highly relevant to fungal biotechnology, ecology, and pathogenicity.

## MATERIALS AND METHODS

### Fungal strains and media.

A list of filamentous fungi strains used in this study is given in Table S1 in the supplemental material. Supplemented minimal medium for A. nidulans and standard strain construction procedures have been described previously (51).

10.1128/mBio.03196-20.2 TABLE S1 Strains used in this study. Download Table S1, DOCX file, 0.01 MB. Copyright © 2021 Fukuda et al. 2021 Fukuda et al. https://creativecommons.org/licenses/by/4.0/ This content is distributed under the terms of the Creative Commons Attribution 4.0 International license.

### Microfluidic device.

The microfluidic devices originally designed for culturing tip-growing plant cells and reported by Yanagisawa et al. (27) were adapted for the current fungal cell studies. Briefly, photoresist (SU-8 3005 and 3010) based microstructures were created on a silicon wafer using a maskless lithography system (DL-1000; Nano System Solutions, Inc.). Then, the polydimethylsiloxane (PDMS, Sylgard 184; Dow Corning) device was prepared through a standard soft-lithography technique. Finally, the PDMS and cover glass (24 × 60 mm, Matsunami) were both treated with O_2_ plasma (CUTE, Femto Science) for permanent bonding.

### Growth condition.

The minimal medium was placed into a 20-ml plastic syringe (SS-20ESZ, Terumo) and infused into the PDMS devices using a positive displacement syringe pump (YSP-101, YMC) at a rate of 0.8 μl per hour through a polyethylene tube (inner diameter 0.38 mm, outer diameter 1.09 mm; BD intramedic).

### Microscopy.

Cells were observed using epifluorescent inverted microscopy, including the Axio Observer Z1 (Carl Zeiss) microscope equipped with a Plan-Apochromat 63× 1.4 oil or 10× or 20× lens objective, an AxioCam 506 monochrome camera, and a Colibri.2 LED light (Carl Zeiss). The temperature of the stage was kept at 30°C by a thermo-plate (TOKAI HIT, Japan). Images were collected and analyzed by the Zen system (Carl Zeiss) and ImageJ software.

### Scanning probe microscopy.

Cells were grown in minimal medium on coverslips at 30°C for 24 h. The medium was removed by pipetting and the cells were analyzed using a scanning probe microscope SPM-9700HT (Shimadzu) with high magnification optical microscope unit, active vibration isolation table, wide area scanner (XY: 125 μm, Z: 5 μm), and fiber light. We used scanning probes (tips), PointProbe-Plus silicon-SPM-Sensor, PPP-NCHAuD (NANOSENSORS), thickness 4.0 ± 1 μm; length 125 ± 10 μm; width 30 ± 7.5 μm; resonance frequency 204-497 kHz; and tip height 10 to 15 μm. Images were collected and analyzed by Nano 3D mapping software (Shimadzu). The nanoindentation results were fitted to the JKR model.

### Statistical methods.

Correlations were measured by CORREL function in Excel. Hierarchical clustering was performed with complete linkage using hclust function in R.
