# Supplementary figures and images for: Trade-off between Plasticity and Velocity in Mycelial Growth
Source: mBio. 2021 Mar 16;12(2):e03196-20. doi: 10.1128/mBio.03196-20 (PMC8092280; doi:10.1128/mBio.03196-20)

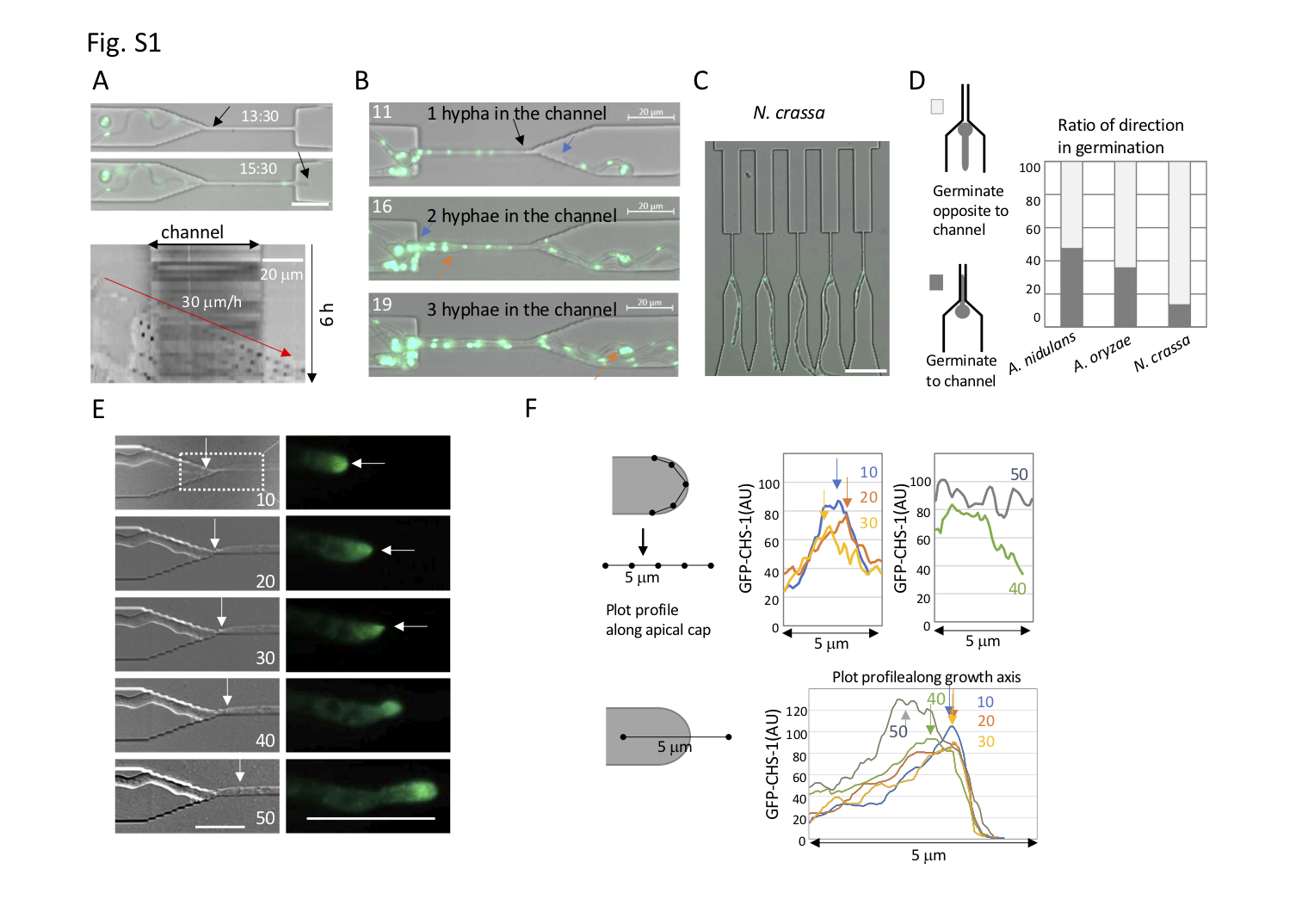

Supplement: FIG S1 [file mBio.03196-20-sf001.tif]
