# Supplementary material for: Trade-off between Plasticity and Velocity in Mycelial Growth
Source: mBio. 2021 Mar 16;12(2):e03196-20. doi: 10.1128/mBio.03196-20 (PMC8092280; doi:10.1128/mBio.03196-20)
Supplement: TABLE S1 [file mBio.03196-20-st001.docx]

| Strain | Genotype | Source |
| --- | --- | --- |
| *Aspergillus nidulans* SRS27 | *gpdA* promoter GFP fused StuA-NLS | 1 |
| *Aspergillus oryzae* RIB40UtH2BG | RIB40Δn (pUtH2BG) | 2 |
| *Neurospora crassa* N22813A | *mat A his-3*^+^::*Pccg-1-hH1*^+^-*sgfp*^+^ | 3 |
| *Neurospora crassa* NES1-15 | *mat A his-3+::Pccg-1::chs-1::sgfp+* | 4 |
| *Fusarium oxysporum* JCM11502 | Wild type | JCM: Japan Collection of Microorganims |
| *Colletotrichum orbiculare* 104-T | Histone H1-GFP | 5 |
| *Rhizopus oryzae* JCM5582 | Wild type | JCM |
| *Coprinopsis cinerea*  #2 + #8 dikaryon | A3B1 + A2B2 | 6, 7 |

1. Suelmann R, Sievers N, Fischer R (1997) Nuclear traffic in fungal hyphae: *in vivo* study of nuclear migration and positioning in *Aspergillus nidulans*. *Mol Microbiol.* **25**:757-769.
2. Yasui M, Oda K, Masuo S, Hosoda S, Katayama T, Maruyama J, Takaya N, Takeshita N (2020) Invasive growth of *Aspergillus oryzae* in rice koji and increase of nuclear number. *Fungal Biol Biotech*. 7:8.
3. Ramos-Garcia SL, Roberson RW, Freitag M, Bartnicki-Garcia S, Mourino-Perez RR (2009) Cytoplasmic bulk flow propels nuclei in mature hyphae of *Neurospora crassa*. *Eukaryot Cel*. **8**:1880-1890
4. Sanchez-Leon E, Verdín J, Freitag M, Roberson RW, Bartnicki-Garcia S, Riquelme M (2011) Traffic of chitin synthase 1 (CHS-1) to the Spitzenkörper and developing septa in hyphae of *Neurospora crassa*: actin dependence and evidence of distinct microvesicle populations. *Eukaryot Cell*. **10**:683-695.
5. Fukada F, Kubo Y (2015) *Colletotrichum orbiculare* regulates cell cycle G1/S progression via a two-component GAP and a GTPase to establish plant infection. *Pant Cell*. **27**:2530-2544.
6. Stajich JE, et al (2010) Insights into evolution of multicellular fungi from the assembled chromosomes of the mushroom *Coprinopsis cinerea* (*Coprinus cinereus*). *Proc Natl Acad Sci USA.* **107**:11889–11894.
7. Masuda R, Iguchi N, Tukuta K, Nagoshi T, Kemuriyama K, Muraguchi H (2016) The *Coprinopsis cinerea* Tup1 homologue Cag1 is required for gill formation during fruiting body morphogenesis. *Biol Open*. **5**:1844-1852.
